# Revealing the Poliovirus’s Path to Infection

**DOI:** 10.1371/journal.pbio.0050205

**Published:** 2007-07-10

**Authors:** Liza Gross

Poliovirus consists of little more than a few genes encased in a protein coat, yet once this RNA virus enters the nervous system, it can induce paralysis within hours. Polio crippled thousands of children a year in industrialized countries during the first half of the 20th century, until the Salk and Sabin vaccines brought the virus under control in the late 1950s and early 1960s. Although five cases of polio were reported in an Amish community in Minnesota in 2005, the disease, which still has no cure, remains endemic in just four countries: Nigeria, India, Pakistan, and Afghanistan.

Like all viruses, the poliovirus’s genetic material sits inside a protective protein-laden “capsid” shell. In most animal viruses, the capsid receives additional protection from a protein-studded viral envelope that facilitates infection by recognizing and binding to receptors on the cell surface. The poliovirus, however, has just the capsid to mediate protection and infection.

Scientists have long debated how the poliovirus manages to get its genome inside the cell. Does it simply inject its RNA through a channel in the plasma membrane or does it commandeer an endocytic pathway, which the cell uses to internalize extracellular molecules? The details of viral entry have long remained obscure, in part because visualizing the process has proven technically difficult and partly because animal viruses produce so many noninfectious particles that confound observations.

In a new study, Boerries Brandenburg, Lily Y. Lee, and colleagues describe a novel approach that circumvents these technical hurdles to track individual virus particles as they infect living cells. Working with the poliovirus as a model for nonenveloped viruses, the researchers used fluorescence microscopy to examine not only how the virus enters the cell, but also where and when it releases its genetic material.

To investigate the poliovirus’s mode of entry, Brandenburg et al. labeled the viral capsid and RNA with distinctly colored fluorescent tags. Getting dyes to bind to viral RNA inside the capsid without inactivating the RNA, however, is no easy feat. The researchers had to screen 20 dyes before they found one that would bind to the RNA as the virus reproduced without compromising the infectivity of the virus. After infecting human cells with the dual-labeled virus, they observed virions releasing their RNA, which the virions did with surprising efficiency: most had been released an hour after infection.

The researchers determined where the RNA was released by analyzing images of the labeled virus at different points after infection. Five minutes after infection, nearly all the virus particles, aglow with both capsid and RNA labels, stayed near the cell surface. By 20–50 minutes after infection, particles gravitated toward the cell interior, most glowing with just the capsid dye. RNA release appeared to occur either near the cell surface or just after entry, and either at the top or bottom surface of the cell.

To find out whether genome release proceeds from the plasma membrane or from discrete compartments (called vesicles) near the membrane, the researchers tagged capsids with a dye that loses fluorescence when exposed to high pH. Capsids outside the cell would be sensitive to pH changes while those internalized would be protected by the cell’s own pH-buffering mechanisms. The percentage of virus particles displaying pH sensitivity decreased over time, suggesting that the particles had entered the cell. And because the dynamics of this process mirrored those of RNA release, and no RNA was released from particles that remained on the cell surface, Brandenburg et al. concluded that RNA release happened after internalization.

**Figure:**
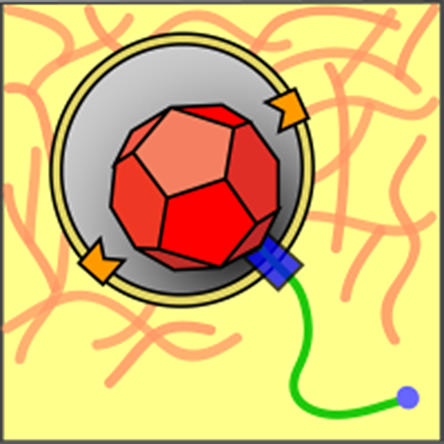
A novel imaging assay reveals how the poliovirus delivers its genome into the host cell.

By using inhibitors designed to disrupt cell processes that might play a role in infection, the researchers revealed that RNA release is not a passive process, but requires energy, just as endocytosis does. Genome release also depends on actin, a structural protein involved in endocytosis. Although infection does not depend on well-known components of the endocytosis pathway, it does require a class of enzymes that interact with the actin network beneath the cell membrane. The dependence of RNA release on various cellular factors determined by this imaging method correlates with the dependence obtained using an infectivity assay, demonstrating that the pathway monitored by imaging is the relevant pathway leading to infection. By testing inhibitors known to target specific enzymes, future studies can determine which one poliovirus hijacks to infect cells.

Altogether, these results suggest that the poliovirus enters the cell after binding to the cell surface, then rapidly releases it genome from vesicles near the membrane. Researchers can now investigate how these vesicles trigger RNA release and how the invasive RNA reaches the cell’s replication machinery. And since nonenveloped viruses often tailor their entry mechanisms to the cell they target, the innovative imaging technique described in this study should provide researchers with a valuable tool to deconstruct, and eventually, prevent their path to infection.

